# Supplementary material for: Exploring collective emotion transmission in face-to-face interactions
Source: PLoS One. 2020 Aug 7;15(8):e0236953. doi: 10.1371/journal.pone.0236953 (PMC7413751; doi:10.1371/journal.pone.0236953)
Supplement: S1 File — (DOCX) [file pone.0236953.s001.docx]

**File S1.** Emotion assessments and pilot study

*Emotion assessments*

Please tick the number that best describes your ratings about the following emotions at this moment with 1= not at all and 7= very strong

| Emotions | not at all |  |  | moderate |  |  | very strong |
| --- | --- | --- | --- | --- | --- | --- | --- |
| Sad | 1 | 2 | 3 | 4 | 5 | 6 | 7 |
| Happy | 1 | 2 | 3 | 4 | 5 | 6 | 7 |
| Disgusted | 1 | 2 | 3 | 4 | 5 | 6 | 7 |
| Excited | 1 | 2 | 3 | 4 | 5 | 6 | 7 |
| Angry | 1 | 2 | 3 | 4 | 5 | 6 | 7 |
| Content | 1 | 2 | 3 | 4 | 5 | 6 | 7 |
| Surprised | 1 | 2 | 3 | 4 | 5 | 6 | 7 |
| Calm | 1 | 2 | 3 | 4 | 5 | 6 | 7 |

*Pilot study.*

Our pilot study was conducted on 40 participants (age range = 11 to 13 years). We used four materials to elicit emotions. All participants read the four materials and rated their emotions on the Positive Affect and Negative Affect Scale (five-point Likert scale). The results are shown in Fig S1, which indicates that the material about movable type printing evoked more negative emotions than the other materials (*F* (3, 111) = 5.837, *p* < .01).
