# Supplementary figures and images for: Exploring collective emotion transmission in face-to-face interactions
Source: PLoS One. 2020 Aug 7;15(8):e0236953. doi: 10.1371/journal.pone.0236953 (PMC7413751; doi:10.1371/journal.pone.0236953)

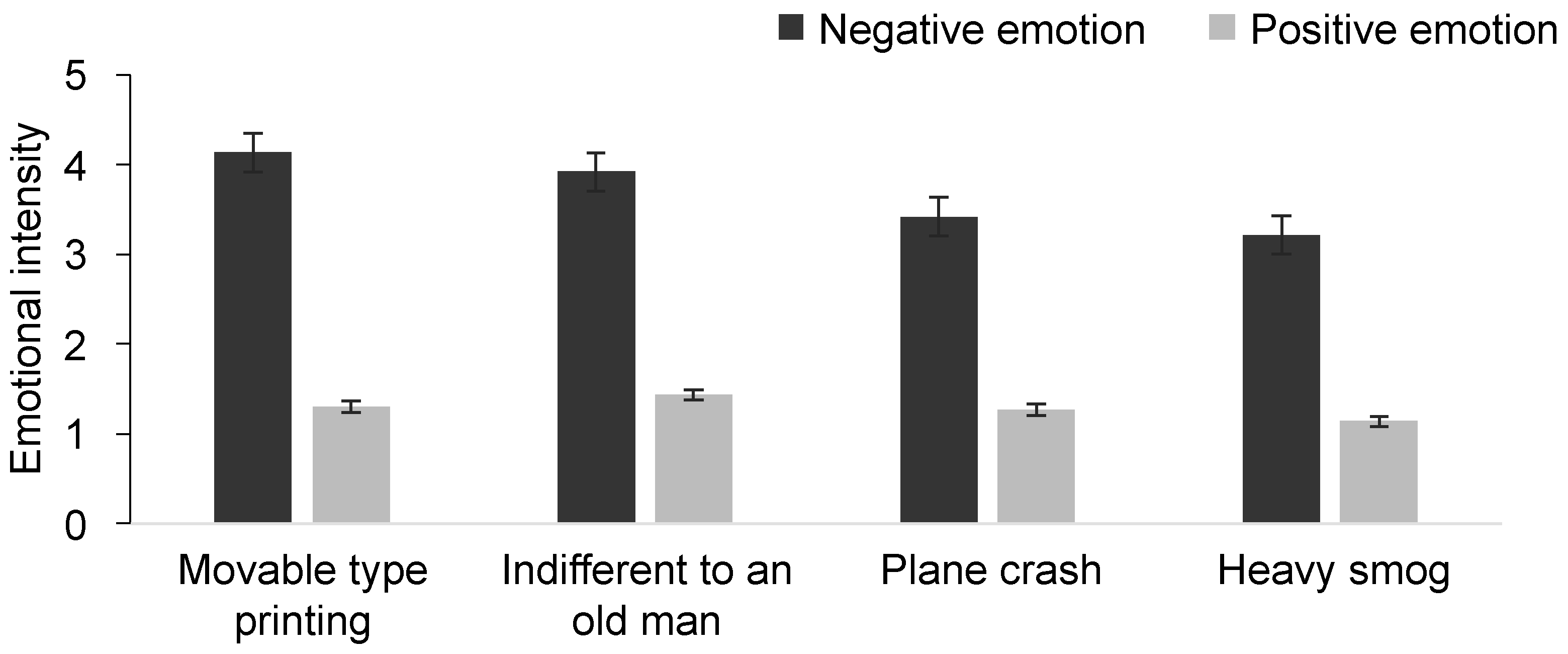

Supplement: S1 Fig — (TIF) [file pone.0236953.s002.tif]
